# Functional Characterization of Plasmid-Borne *rmpADC* Homologues in Klebsiella pneumoniae

**DOI:** 10.1128/spectrum.03081-22

**Published:** 2023-04-24

**Authors:** Xuemei Yang, Xiaoxuan Liu, Edward Wai-Chi Chan, Rong Zhang, Sheng Chen

**Affiliations:** a Department of Food Science and Nutrition, Faculty of Science, The Hong Kong Polytechnic University, Hung Hom, Hong Kong; b Department of Infectious Diseases and Public Health, Jockey Club College of Veterinary Medicine and Life Sciences, City University of Hong Kong, Kowloon, Hong Kong; c State Key Lab of Chemical Biology and Drug Discovery, Department of Applied Biology and Chemical Technology, The Hong Kong Polytechnic University, Hung Hom, Hong Kong; d Department of Clinical Laboratory, Second Affiliated Hospital of Zhejiang University, School of Medicine, Hangzhou, Zhejiang, China; Yangzhou University

**Keywords:** *Klebsiella pneumoniae*, *rmpADC* homologues, virulence plasmid, capsule, hypermucoviscosity

## Abstract

Expression of the hypermucoviscosity (HMV) phenotype and capsular polysaccharide (CPS) biosynthesis in Klebsiella pneumoniae were reported to be encoded by genes located in the chromosomal *rmp* locus. However, the functions of the *rmp* locus in the virulence plasmid remained unclear, and most of the *rmp* loci in clinical K. pneumoniae are plasmid carried. In this study, we investigated the functional characteristics of plasmid-borne *rmp* homologues in clinical hypervirulent K. pneumoniae (hvKP) strains by cloning and introducing such gene homologues into K. pneumoniae strains of different capsule types, followed by the evaluation of phenotypic changes in these strains. Acquisition of the plasmid-borne p*rmpADC* and p*rmpA2D2* loci were found to result in an increase in mucoviscosity and CPS production in K1 and K2 K. pneumoniae, while only the p*rmpA2D2* locus contributed to phenotypic changes in the ST11/KL64 strain. Consistently, both *rmpD* and *rmpD2* increased HMV in K1 and K2 K. pneumoniae, while only *rmpD2* contributed to HMV in the ST11/KL64 strain; *rmpC* contributed to CPS overproduction in K1 and K2 strains but not in the ST11/KL64 strain. Furthermore, we proposed a logistic molecular basis of the HMV phenotype of K. pneumoniae on which p*rmpD2*-mediated HMV is attributed to the increase of cell-free CPS production. Our data confirm that the *rmp* homologues carried by the virulence plasmid play a key role in virulence expression in K. pneumoniae, but the phenotype is highly dependent on the genetic background of the host strain and explained why most of the clinical ST11 strains carry only the p*rmpA2D2* locus.

**IMPORTANCE** Klebsiella pneumoniae has become the most frequently isolated bacterial pathogen in hospital settings, with a very high mortality rate worldwide. Factors contributing to the virulence of K. pneumoniae are the overproduction of capsular polysaccharide (CPS) as well as the hypermucoviscosity (HMV) phenotype. These two phenotypes were reported to be regulated by *rmpA/A2* homologues, which are often carried by virulence plasmids. Here, we determined the functional role of two plasmid-borne *rmpA* in mediating expression of the HMV phenotype and CPS production in K. pneumoniae. Different capsule types exhibited differences in the expression of HMV and CPS production although they harbored an identical plasmid-borne *rmpA* or *rmpA2* locus, indicating that these virulence-related phenotypes are strongly related to the genetic background of the host strains. Our study provides a novel understanding of the regulation of virulence-related phenotypes and clinical management of K. pneumoniae infections.

## INTRODUCTION

Klebsiella pneumoniae is a human commensal and opportunistic pathogen that can cause severe hospital-acquired infections, especially among patients with a compromised immune system (1). In recent years, strains that exhibit a variety of drug resistance and virulence phenotypes have emerged, posing further challenges to the clinical treatment of infections caused by this important pathogen. Unlike the classical K. pneumoniae (cKP) strains, hypervirulent K. pneumoniae (hvKP) strains are less commonly associated with antibiotic resistance; such strains are considered community acquired as they cause infections in healthy hosts that manifest as pyogenic liver abscesses (2, 3). Two features known to distinguish cKP from hvKP are the number of siderophore systems and the abundance of the capsular polysaccharide (CPS) that they harbor (1). Classical strains typically have one or two siderophore systems, whereas hvKP strains have three or four. In addition, hvKP strains are known to produce a very thick CPS which is associated with a hypermucoid phenotype known as hypermucoviscosity (HMV) (4). These functions are encoded by genes located in the virulence plasmid, which include the *iuc* (biosynthetic genes for the siderophore aerobactin), *iro* (biosynthetic genes for the siderophore salmochelin), and *rmpA* and *rmpA2* (regulators of the mucoid phenotype) genes (5–8).

The HMV phenotype of K. pneumoniae was previously thought to be due to the high-level abundance of CPS in hvKP. However, recent studies demonstrated that, although expression of the HMV phenotype and CPS biosynthesis are closely related, they could be dissociable and each served distinct functions during pathogenesis (9). Walker et al. identified an *rmp* operon that comprised the *rmpA*, *rmpD*, and *rmpC* genes in strain KPPR1S, in which the *rmpA* gene product autoregulates the operon, *rmpD* confers the HMV phenotype, and *rmpC* promotes CPS biosynthesis (10, 11). However, the *rmp* locus of strain KPPR1S is located in the integrative and conjugative element (ICE) ICE*Kp1* in the chromosome, which is not a representative of the *rmp* homologues present in the majority of hvKP strains (12). The *rmpA* and *rmpA2* genes are more frequently associated with the virulence plasmid (13, 14). Furthermore, the virulence plasmid has undergone evolution to generate variants that carried only one of these regulators, as well as those which carried mutated *rmpA*/*rmpA2* genes (15, 16). However, the impact of these evolution processes on the virulence level of K. pneumoniae remained unknown. In this study, we investigated the functional features of *rmp* homologues in clinical hvKP strains by cloning and introducing various *rmp* homologues into clinical K. pneumoniae strains of various capsule types and assessed the level of virulence that they encoded. Our data will provide in-depth understanding of the roles of newly evolved plasmid-borne *rmp* homologues in virulence expression in clinical K. pneumoniae strains and hence provide important insights into the development of effective approaches to treat hvKP infections.

## RESULTS

### Plasmid-borne *rmpADC* homologues in clinical K. pneumoniae strains.

**FIG 1:**
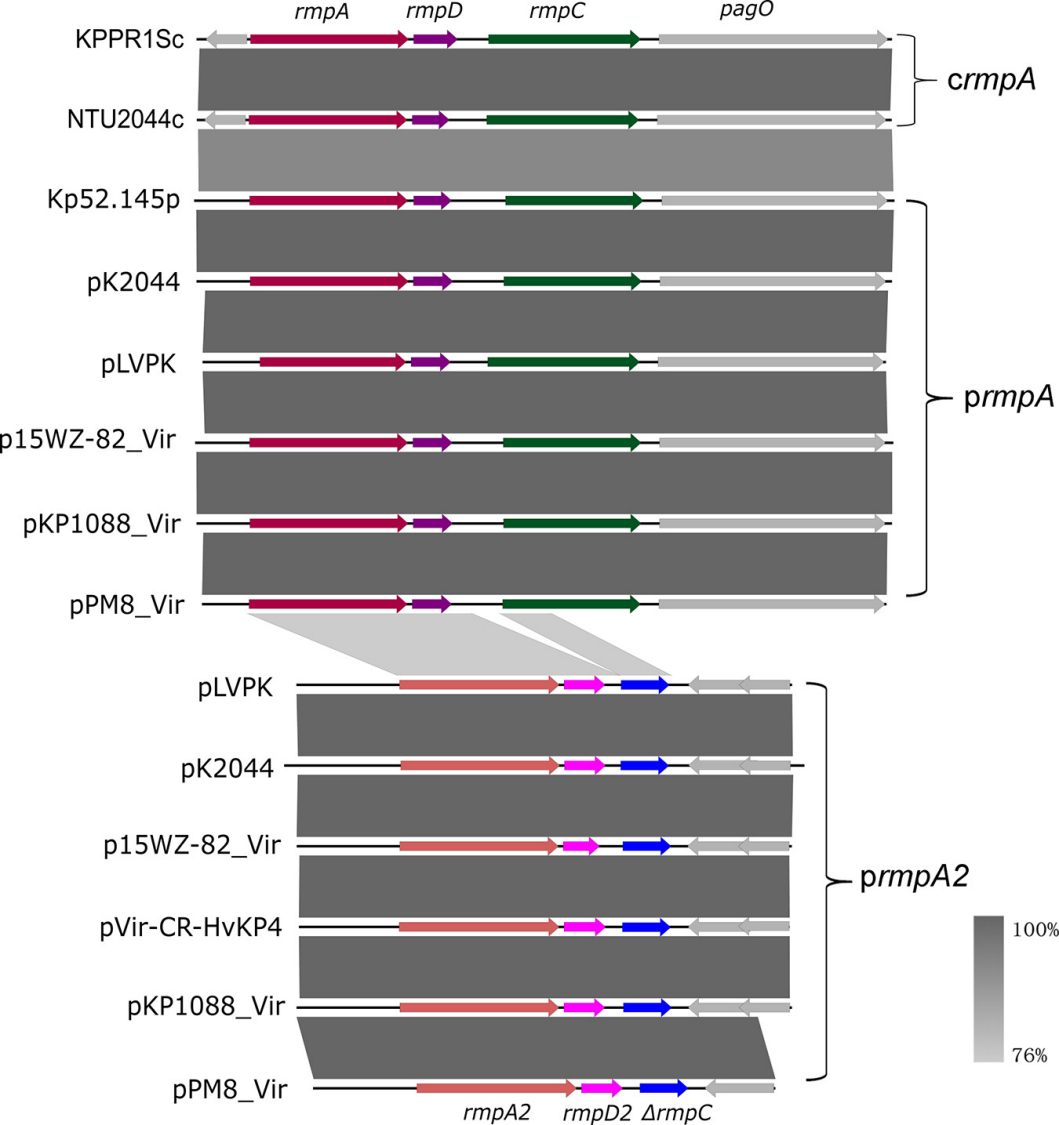
Alignment of the c*rmpA*, p*rmpA*, and p*rmpA2* loci in clinical K. pneumoniae strains. There were two *rmpA*/*rmpA2* genes in the large virulence plasmids pK2044 and pLVPK (6). The p*rmpA* locus was found to comprise the *rmpA*, *rmpD*, and *rmpC* genes, resembling the c*rmp* locus of strains KPPR1S and NTU2044, with 90% sequence identity, whereas the p*rmpA2* locus contained the *rmpA2*, *rmpD2*, and truncated *rmpC* genes and shared 78% sequence identity with the c*rmpA* and p*rmpA* loci.

**FIG 2:**
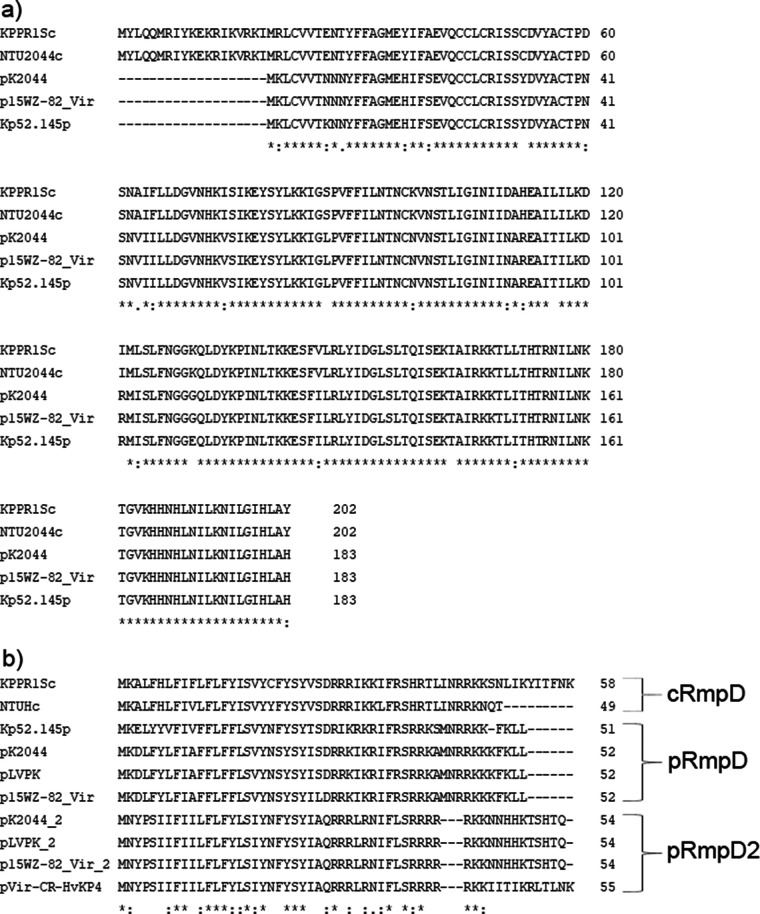
Amino acid sequence alignment of gene products of the *rmpC* (a) and *rmpD* (b) homologues located in the chromosome and plasmid, respectively. Below the protein sequences is a key denoting conserved sequence (*), conservative mutations (:), semi-conservative mutations (.), and non-conservative mutations ( ).

The *rmp* locus was mainly located in the ICE*Kp1* element in the chromosome of K. pneumoniae but more commonly in large virulence plasmids. It was reported that the chromosomal *rmp* locus of strain KPPR1S comprised the *rmpA*, *rmpD*, and *rmpC* genes, which played different roles in the expression of the HMV phenotype and CPS biosynthesis (10, 11). We then analyzed the *rmp* homologues in the virulence plasmids and determined whether their functional roles resembled that of c*rmp*, since the plasmid-borne *rmp* locus is much more common in clinical K. pneumoniae isolates than those located in the chromosome. There were two *rmpA*/*rmpA2* genes in the large virulence plasmids, namely, pK2044 and pLVPK, each located in a separate genetic locus (6). The p*rmpA* locus was found to contain the *rmpA*, *rmpD*, and *rmpC* genes, resembling the c*rmp* locus of strains KPPR1S and NTU2044, with 90% sequence identity. The p*rmpA2* locus comprised the *rmpA2* and *rmpD* genes, and a truncated *rmpC* gene, sharing 78% sequence identity with the c*rmpA* and p*rmpA* loci (Fig. 1). For the sake of differentiation, the *rmpD* homologue located downstream of the *rmpA2* gene was designated *rmpD2*. The *rmpC* genes were conserved among the c*rmp* and p*rmp* loci. The product of the p*rmpC* gene from plasmid p15WZ-82_Vir exhibited 88% sequence identity with the product of the c*rmpC* gene from the chromosome of strain KPPR1S (Fig. 2a). The *rmpD* genes were more variable. The product RmpD2 from plasmid pVir-CR-HvKP4 exhibited 58% sequence identity with pRmpD from plasmid pLVPK and 52% sequence identity with cRmpD from the chromosome of strain KPPR1S (Fig. 2b). The plasmids p15WZ-82_Vir and pVir-CR-HvKP4 reported in our previous studies were able to mediate the expression of HMV and virulence, as well as the overproduction of CPS in K. pneumoniae strains (17, 18). Plasmid p15WZ-82_Vir was found to contain both the p*rmpADC* and the p*rmpA2D2* loci, whereas plasmid pVir-CR-HvKP4 carried only the p*rmpA2D2* locus (Fig. 1). However, the p*rmpA2* gene in plasmid p15WZ-82_Vir was mutated in such a way that it encoded a truncated protein. These findings prompted us to further study the role of these plasmids in mediating the onset of hypervirulence phenotypes in K. pneumoniae. It should be noted that all K1/K2 strains and an increasing number of non-K1/K2 clinical HvKP strains that harbor a virulence plasmid contain both the p*rmpADC* and the p*rmpA2D2* loci.

### The *rmpADC* homologues mediate expression of hypermucoviscosity and CPS overproduction in clinical K. pneumoniae strains.

**FIG 3:**
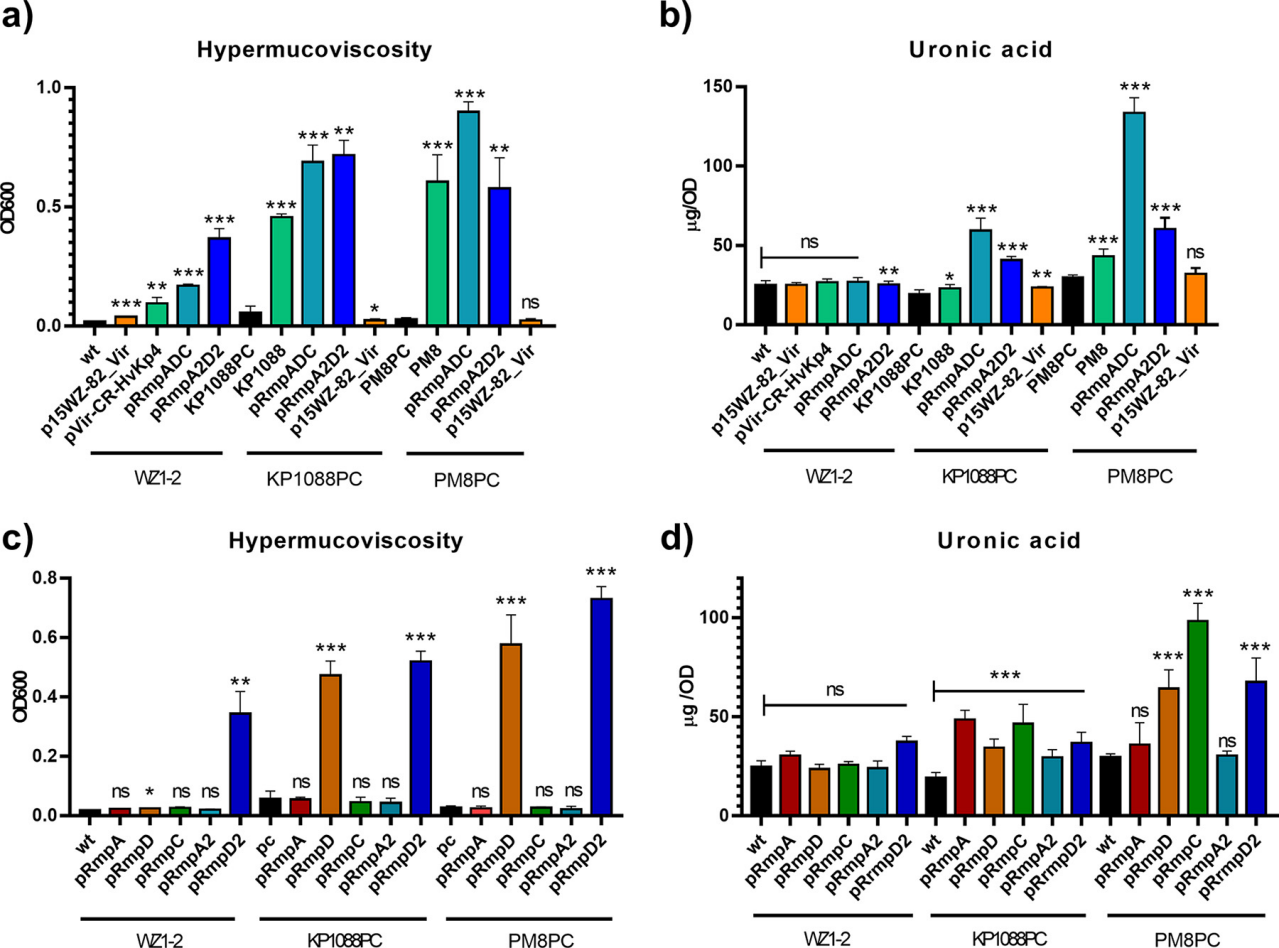
Mucoviscosity assay and uronic acid assay of ST11 CRKP strain WZ1-2, K1 strain KP1088, and K2 strain PM8 carrying different virulence determinants. Mucoviscosity (a) and uronic acid production (b) of strain WZ1-2 and its variants receiving virulence plasmid p15WZ-82_Vir, pVir-CR-HvKP4, the p*rmpADC* and p*rmpA2D2* loci, strain KP1088, its virulence plasmid curing variant KP1088PC, and KP1088PC receiving the p*rmpADC* and p*rmpA2D2* loci, and strain PM8, its virulence plasmid curing variant PM8PC, and PM8PC receiving the p*rmpADC* and p*rmpA2D2* loci. Mucoviscosity (c) and uronic acid production (d) of strains WZ1-2, KP1088PC, and PM8PC with their variants receiving p*rmpA*, p*rmpD*, p*rmpC*, p*rmpA2*, and p*rmpD2*. Virulence plasmids p15WZ-82_Vir and pVir-CR-HvKP4 were conjugated to strain WZ1-2. wt, wild type; ns, not significant. Each value is presented as the mean, and error bar indicates SD. *, *P* < 0.05, **, *P* < 0.01, ***, *P* < 0.001, ns, *P* ≥ 0.05, wt, wild type strain.

To further determine the functional role of p*rmpA* and p*rmpA2*, these two genetic loci were cloned and ligated to vector pCE2 and then transformed into different K. pneumoniae strains. Introduction of the virulence plasmid p15WZ-82_Vir or pVir-CR-HvKP4, as well as the p*rmpA* or p*rmpA2* locus, into strain WZ1-2 could in each increase the mucoviscosity (Fig. 3a). However, none of these genetic elements caused a significant increase in CPS production in strain WZ1-2 (Fig. 3b). Furthermore, as the HMV phenotype has been primarily associated with K1 and K2 serotypes, we also included a K1 K. pneumoniae strain, KP1088, and a K2 K. pneumoniae strain, PM8, as hosts in the functional study of the p*rmpA* and p*rmpA2* clusters. Strains KP1088 and PM8 both carried a pLVPK-like virulence plasmid with p*rmpA* and p*rmpA2* clusters (Fig. 1). The original virulence plasmids harbored by the strains KP1088 and PM8 were cured, and the plasmid-cured strains KP1088PC and PM8PC were found to exhibit low mucoviscosity and low-level CPS production (Fig. 3a and b). Both the p*rmpA* and p*rmpA2* clusters were found to cause a significant increase in mucoviscosity and CPS production in strains KP1088PC and PM8PC (Fig. 3a and b), even though *rmpC* is not present in the p*rmpA2* cluster. Introduction of the whole plasmid p15WZ-82_Vir, however, has a rather limited effect on these two phenotypes in these three tested strains. It is worth noting that the p*rmpA* cluster exhibited a more prominent effect in virulence expression in clinical K1 strain KP1088PC and K2 strain PM8PC, whereas the effect of the p*rmpA2* cluster was more apparent in KL64 strain WZ1-2.

### Regulation of hypermucoviscosity and CPS overproduction in clinical K. pneumoniae strains.

To further investigate the role of the p*rmpA* and p*rmpA2* loci in mediating expression of the virulence-related phenotypes, the genes p*rmpA*, p*rmpD*, p*rmpC*, p*rmpA2*, and p*rmpD2* were individually amplified and introduced into strains WZ1-2, KP1088PC, and PM8PC, separately. The results showed that only the p*rmpD* and p*rmpD2* genes were able to encode the HMV phenotype (Fig. 3c); this finding is consistent with those of previous studies (11). To rule out the possibility that the HMV phenotype is the result of a high copy number of *rmpD*, a low-copy-number vector, pSC101, was ligated to the target gene and transformed into strain PM8PC. As Fig. S1 in the supplemental material shows, HMV was obtained in PM8PC/rmpD-pSC101, suggesting that a high copy number of *rmpD* is not a necessity for HMV. Furthermore, our results indicate that p*rmpD* caused a significant increase in mucoviscosity in the K1 strain KP1088PC and the K2 strain PM8PC, but not the ST11/KL64 strain WZ1-2. However, p*rmpD2* was found to cause a dramatic increase in mucoviscosity in all these three strains (Fig. 3c). These data indicate that p*rmpD2* is functionally better adapted in K. pneumoniae isolates of various capsule types, including ST11 carbapenem-resistant K. pneumoniae (CRKP). Regulation of CPS production is much more complicated. Previous studies showed that c*rmpA* and c*rmpC*, but not c*rmpD*, regulated CPS expression in K2 strain KPPR1S (10). Although each of the p*rmpA*, p*rmpD*, p*rmpC*, p*rmpA2*, and p*rmpD2* genes was found to cause upregulation of CPS production in K1 strain KP1088PC, only p*rmpD*, p*rmpC*, and p*rmpD2* could encode a significant increase in CPS production in K2 strain PM8PC (Fig. 3d). No significant changes in the level of CPS production were observed after introduction of both the p*rmpADC* and p*rmpA2D2* loci, or the genes p*rmpA*, p*rmpD*, p*rmpC*, p*rmpA2*, and p*rmpD2*, into ST11/KL64 strain WZ1-2 (Fig. 3b and d). Our data confirmed that both pRmpD and pRmpD2 in the virulence plasmid could mediate the expression of the HMV phenotype in a manner similar to cRmpD (11). Furthermore, p*rmpD* and p*rmpD2* also contributed to an increase in CPS production in K1 and K2 strains. Like c*rmpC*, p*rmpC* caused overproduction of CPS in K1 and K2 strains but not ST11/KL64 strain WZ1-2.

### Correlation of hypermucoviscosity with CPS production.

**FIG 4:**
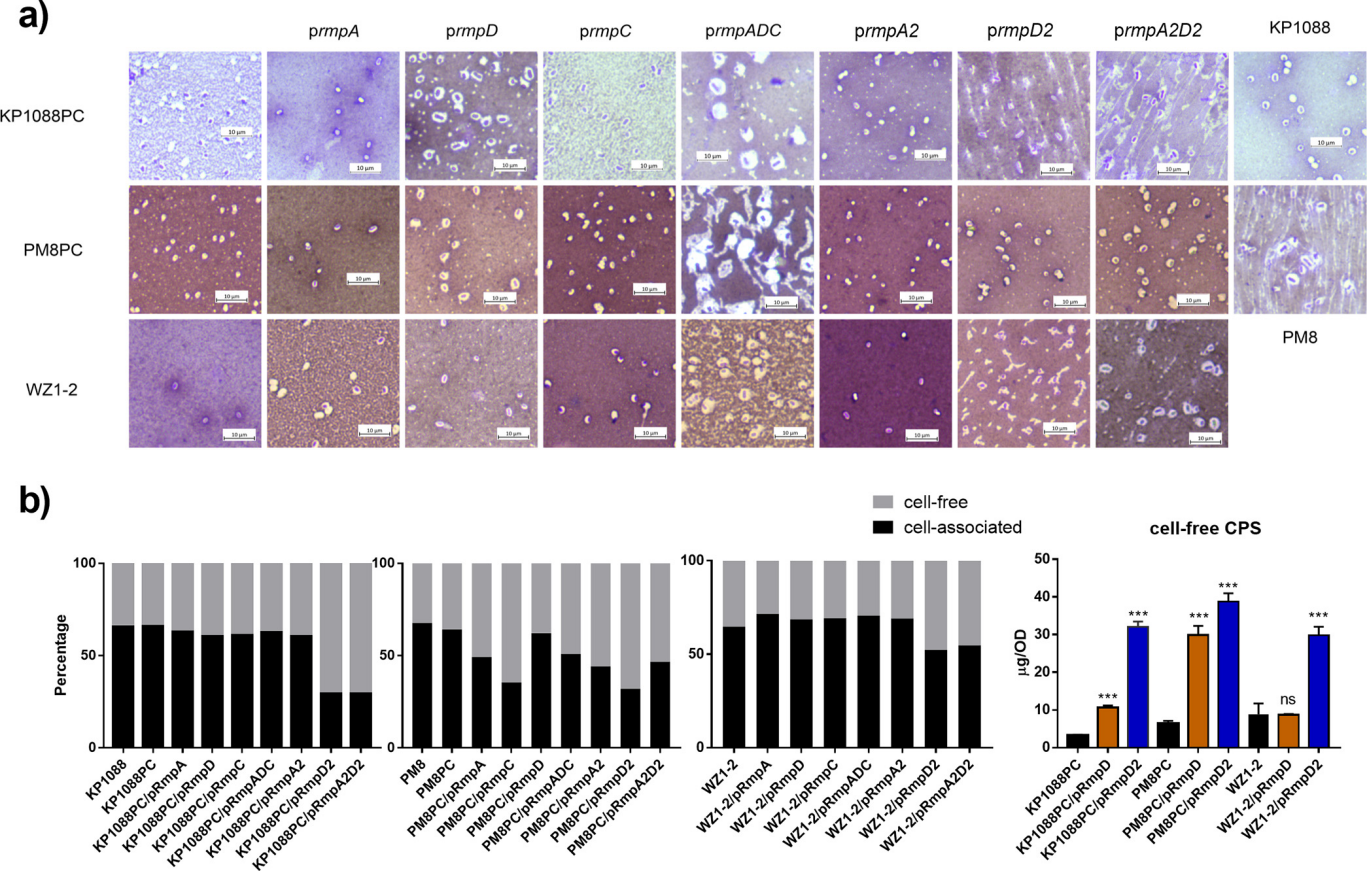
Capsule staining and proportion of extracellular and cell surface CPS of ST11 CRKP strain WZ1-2, K1 strain KP1088PC, and K2 strain PM8PC, which carried the virulence determinants of p*rmpA*, p*rmpC*, p*rmpD*, p*rmpADC*, p*rmpA2*, p*rmpD2*, and p*rmpA2D2.* (a) Increased thickness of capsule coat was observed in K1 and K2 strains carrying p*rmpD* and p*rmpADC* and the ST11 strain carrying p*rmpADC*, while not in variants carrying the p*rmpA2* locus of any capsule types. (b) RmpD2-regulated increase of cell-free CPS was conserved in K1, K2, and ST11 CRKP strains.

Next, we tried to visualize the capsule of variants harboring different genetic elements by Indian ink staining. The nonmucoid strains, such as CRKP strain WZ1-2 and the plasmid-cured strains KP1088PC and PM8PC, exhibited a thin-layered capsule, whereas the corresponding hypermucoid variants, such as KP1088PC/pRmpD and PM8PC/pRmpD, exhibited a large halo around the cell, indicating the presence of a much thicker capsule (Fig. 4a). However, a significant increase in capsule size could not be observed in variants that produced a larger amount of CPS without exhibiting HMV, such as KP1088PC/pRmpC and PM8PC/pRmpC (Fig. 4a). Variants that exhibited both HMV and CPS overproduction, such as KP1088PC/pRmpADC and PM8PC/pRmpADC, possessed the capsule of the largest size, suggesting that HMV and overproduced CPS resulted in the formation of a superthick capsule (Fig. 4a). However, it should be noted that this phenotype was observed only in variants that have acquired the p*rmpADC* locus, but not the p*rmpA2D2* locus.

One hypothesis is that the HMV phenotype is correlated with cell-associated CPS. Previous studies on Streptococcus pneumoniae demonstrated that compared to other serotypes, hypermucoid serotype 3 isolates synthesize CPS by the synthase-dependent pathway, resulting in CPS release during growth *in vivo* and *in vitro* (19). Hence, we quantified the cell-released CPS and cell-associated CPS during *in vitro* growth of different K types and their variants. What was consistent among all three capsule types tested was that pRmpD2 drastically increased the releases of CPS into the culture medium, causing the cell-free CPS to be the majority of total CPS (Fig. 4b). These findings suggested that HMV correlated with cell-released CPS, rather than total CPS.

## DISCUSSION

In this study, we determined the functional roles of two plasmid-borne *rmpA* homologues in mediating the expression of the HMV phenotype and CPS production in K. pneumoniae. The tested strains included a clinical ST11/KL64 CRKP strain, a virulence plasmid-cured K1 strain, and a virulence plasmid-cured K2 strain. The p*rmpA* locus comprised the p*rmpA*, p*rmpD*, and p*rmpC* genes, resembling the c*rmp* locus of strains KPPR1S and NTU2044. The p*rmpA2* locus comprised the p*rmpA2* and p*rmpD2* genes, as well as a truncated p*rmpC* gene. Our results showed that the two gene clusters produced similar phenotypes in K1 and K2 K. pneumoniae strains, although the p*rmpA* locus was slightly more effective in upregulating the expression of HMV and CPS production in both K1 and K2 strains. In KL64 strain WZ1-2, the p*rmpA2* cluster encoded a more viscous phenotype than did the p*rmpA* cluster. We reported for the first time that different capsule types exhibited differences in the expression of HMV and CPS production even though they harbored an identical p*rmpA* or p*rmpA2* locus. The finding that both the p*rmpADC* and p*rmpA2D2* loci contributed to increase of the mucoviscosity and CPS production in K1 and K2 strains, but only an increase of mucoviscosity in the KL64 strain, is intriguing.

The c*rmpD* gene was previously reported to be a key factor underlying HMV expression in K2 strain KPPR1S (11). Here, we confirm that the functional role of p*rmpD* and p*rmpD2* from the virulence plasmids in K1 and K2 strains is similar to that of their chromosomal counterpart. These observations are consistent with that of a previous report on clinical non-K1/K2 CR-HvKP strains that carried a virulence plasmid without the *rmpA* locus (15). Both the *rmpA* and *rmpC* genes were reported to regulate CPS biosynthesis (6, 10), yet we observed the regulatory effects of p*rmpA* and p*rmpA2* in the K1 strain only and observed that p*rmpC* played a regulatory role in both K1 and K2 strains, indicating that the impact of products of these genes on the phenotypes of HMV and CPS production is strongly dependent on the genetic background of the host strains, especially the capsule type.

Furthermore, our data suggested that the HMV phenotype correlated with the release of CPS into medium during growth. A higher CPS ratio in the culture supernatant than on the bacterial surface can lead to a higher antibody-absorbing capacity by the CPS in the culture supernatant (19). Therefore, lower efficiency might occur when anti-CPS vaccines are used for the treatment of hypermucoid K. pneumoniae. On the one hand, released CPS can absorb free unbound antibodies; meanwhile, antibody-bound CPS could also be released from bacteria, resulting in ineffective immunotherapy.

One limitation of this study is that the impact of p*rmpADC* and p*rmpA2D2* loci on virulence in different genetic backgrounds of K. pneumoniae strains *in vivo* has not been investigated. The cRmpD has been confirmed to contribute to immune evasion and virulence in a K1 K. pneumoniae strain (11), while the virulence of these components in different genetic backgrounds (K types) of K. pneumoniae remains unknown. This work calls for further studies on the investigation of these aspects. The results will provide new information on this emerging pathogen and provide insights for the development of new therapeutic strategies. Also, the molecular basis of HMV needs to be further elucidated.

## MATERIALS AND METHODS

### Bacterial strains, plasmids, and growth conditions.

The strains and plasmids used in this work are listed in Table 1. Recombinant plasmids were introduced into Escherichia coli and K. pneumoniae strains by heat shock at 42°C. Virulence plasmids p15WZ-82_Vir and pVir-CR-HvKP4 were introduced into K. pneumoniae strains by conjugation. E. coli and K. pneumoniae strains were grown in Luria-Bertani (LB) broth at 37°C. Kanamycin (Kan) was added to the culture medium at a concentration of 50 μg/mL appropriately.

**TABLE 1:** Strains and plasmids used in this work

Strain or plasmid	Relevant genotype	Source or reference
Strains		
E. coli DH5α	F^−^ ϕ80*lac*ZΔM15 Δ(*lacZYA*-*argF*)*U169 recA1 endA1 hsdR17*(r_K_^−^ m_K_^+^) *phoA supE44 thi*-*1 gyrA96 relA1* λ^−^	Invitrogen
K. pneumoniae		
CR-HvKP4	CR-HvKP, ST11, KL47	18
15WZ-82	Klebsiella variicola with a conjugative virulence plasmid	17
WZ1-2	CRKP, ST11, KL64	23
WZ1-2/pVir-CR-HvKP4	WZ1-2 received virulence plasmid pVir-CR-HvKP4	This study
WZ1-2/p15WZ-82_Vir	WZ1-2 received virulence plasmid p15WZ-82_Vir	This study
KP1088PC	K1 with virulence plasmid cured	This study
PM8PC	K2 with virulence plasmid cured	This study
Plasmids		
pCE2	Amp^r^ Kan^r^; pUC ori TA cloning vector; *ccdB*	Vazyme
pCE2/RmpADC	*rmpADC* in pCE2	This study
pCE2/RmpA	*rmpA* in pCE2	This study
pCE2/RmpD	*rmpD* in pCE2	This study
pCE2/RmpC	*rmpC* in pCE2	This study
pCE2/RmpA2D2	*rmpA2D2* in pCE2	This study
pCE2/RmpA2	*rmpA2* in pCE2	This study
pCE2/RmpD2	*rmpD2* in pCE2	This study

### Cloning of plasmid-borne *rmp* homologues.

The p*rmpA* and p*rmpA2* loci were amplified using genomic DNA of strains 15WZ-82 and CR-HvKP4 as the templates, respectively. Briefly, fragments of 200-bp sequences upstream of the *rmpA* gene and downstream of the *rmpC* gene were amplified and ligated to vector pCE2 by TA cloning and then transformed into E. coli strain DH5α by heat shock at 42°C. Cloning of the single gene from the p*rmpA* and p*rmpA2* loci followed the same procedure as that used in the cloning of the loci. The resulting plasmids recovered from the transformants were verified by sequencing and then transformed into K. pneumoniae strains WZ1-2, KP1088PC, and PM8PC, respectively. The primers used for cloning are listed in Table S1 in the supplemental material.

### Conjugation assay.

Virulence plasmids p15WZ-82_Vir and pVir-CR-HvKP4 were introduced into K. pneumoniae strain WZ1-2 by conjugation as previously described (17). E. coli transconjugants EC600/15WZ-82-TC and EC600/CR-HvKP4-TC harboring virulence plasmids p15WZ-82_Vir and pVir-CR-HvKP4, respectively, in our previous studies were treated as donor strains (17, 20). Transconjugants of strain WZ1-2 were selected on MacConkey agar plates containing 8 μg/mL potassium tellurite (Te) and 2 μg/mL meropenem. Carriage of *rmpA* or *rmpA2* as a marker gene of the virulence plasmids in transconjugants was determined by PCR.

### Virulence plasmid curing.

The virulence plasmids of K. pneumoniae strains KP1088 and PM8 were cured using sodium dodecyl sulfate (SDS) (21). Briefly, an inoculum of 100 μL overnight culture was added into LB broth containing 5%, 4%, 3%, 2%, and 1% SDS, respectively. The cultures were incubated with shaking at room temperature for 48 h and then diluted and spread on LB agar plates for selection of virulence plasmid-cured colonies. Single colonies were streaked on LB agar plates with or without 10 μg/mL potassium tellurite (Te). Colonies that could grow on the LB agar plate instead of the Te plate were further tested for the presence of the *rmpA2* gene by PCR, and those which were negative for *rmpA2* were regarded as plasmid-cured strains.

### Mucoviscosity assay.

The mucoviscosity of the test strains was determined using the sedimentation assay with slight modifications (17). Briefly, an overnight bacterial culture was normalized to an optical density (OD) of 1.0 and centrifuged for 5 min at 1,000 × *g*. The supernatant was gently removed without disturbing the pellet for measurement of OD at 600 nm (OD_600_). Results were presented as mean and standard deviation from data of three independent experiments. An unpaired two-sided Student *t* test was performed to analyze the statistical difference between the level of mucoviscosity of wild-type strains and that of transformants carrying different *rmp* genes, as well as transconjugants carrying the virulence plasmids. Analysis of data was performed by using GraphPad Prism 8 (San Diego, CA). The string test was performed by stretching bacterial colonies on a sheep blood agar plate using an inoculation loop.

### CPS production.

Uronic acid was extracted and quantified as described previously (17). One milliliter of the overnight culture which had been normalized to an OD of 1.0 in the mucoviscosity assay was centrifuged at 14,000 rpm for 15 min to separate the supernatant and the cell pellet. The cell pellet was resuspended in 1 mL fresh LB for quantification. The normalized culture was represented as total CPS, and the supernatant and the cell pellet were represented as cell-free and cell-associated CPS, respectively. Five hundred microliters of normalized culture (OD = 1.0), supernatant, and cell pellet was mixed with 100 μL of 1% Zwittergent 3-14 in 100 mM citric acid, respectively, followed by incubation at 50°C for 20 min. The cells were then pelleted by centrifugation for 10 min at 14,000 rpm, and 300 μL of the supernatant was collected and added to 1.2 mL of absolute ethanol, followed by incubation at 4°C for 20 min and then centrifugation for 5 min at 14,000 rpm. The pellet was dried and resuspended in 200 μL of distilled water, to which 1.2 mL of 12.5 mM sodium tetraborate in sulfuric acid was added, followed by incubation at 100°C for 5 min and then on ice for 10 min. A 20-μL volume of 0.15% 3-phenylphenol in 0.5% NaOH was then added. After incubation for 5 min at room temperature, the absorbance at 520 nm was measured. The glucuronic acid content of the mixture was determined by extrapolating from a standard curve of glucuronic acid and expressed as micrograms per OD unit. Results were presented as mean and standard deviation from data of three independent experiments. An unpaired two-sided Student *t* test was performed to analyze the statistical difference as described above.

### Capsule staining.

The capsule of the test strains was visualized by negative staining with Indian ink, followed by counterstaining of the bacterial cells with crystal violet (22). Briefly, a loopful of overnight bacterial culture was mixed with a single drop of Indian ink placed on a clean microscope slide. The mixture was gently spread out on the slide using a coverslip and allowed to air dry. The film was then flooded with 0.1% crystal violet for 10 s and gently rinsed with distilled water. The slide was air dried and covered with a coverslip, observed, and photographed under microscope with ×1,000 magnification. Scale bars were added by ZEN.34 (ZEN Lite), and the image of the region of interest was captured by ImageJ.

## References

[1] Paczosa MK, Mecsas J. 2016. *Klebsiella pneumoniae*: going on the offense with a strong defense. Microbiol Mol Biol Rev 80:629–661. doi:10.1128/MMBR.00078-15. 27307579 PMC4981674

[2] Shon AS, Bajwa RPS, Russo TA. 2013. Hypervirulent (hypermucoviscous) Klebsiella pneumoniae: a new and dangerous breed. Virulence 4:107–118. doi:10.4161/viru.22718. 23302790 PMC3654609

[3] Siu LK, Yeh K-M, Lin J-C, Fung C-P, Chang F-Y. 2012. Klebsiella pneumoniae liver abscess: a new invasive syndrome. Lancet Infect Dis 12:881–887. doi:10.1016/S1473-3099(12)70205-0. 23099082

[4] Martin RM, Bachman MA. 2018. Colonization, infection, and the accessory genome of *Klebsiella pneumoniae*. Front Cell Infect Microbiol 8:4. doi:10.3389/fcimb.2018.00004. 29404282 PMC5786545

[5] Russo TA, Olson R, Fang C-T, Stoesser N, Miller M, MacDonald U, Hutson A, Barker JH, La Hoz RM, Johnson JR, Backer M, Bajwa R, Catanzaro AT, Crook D, de Almeda K, Fierer J, Greenberg DE, Klevay M, Patel P, Ratner A, Wang J-T, Zola J. 2018. Identification of biomarkers for differentiation of hypervirulent *Klebsiella pneumoniae* from classical *K. pneumoniae*. J Clin Microbiol 56:e00776-18. doi:10.1128/JCM.00776-18. 29925642 PMC6113484

[6] Hsu C-R, Lin T-L, Chen Y-C, Chou H-C, Wang J-T. 2011. The role of Klebsiella pneumoniae rmpA in capsular polysaccharide synthesis and virulence revisited. Microbiology (Reading) 157:3446–3457. doi:10.1099/mic.0.050336-0. 21964731

[7] Nassif X, Fournier JM, Arondel J, Sansonetti PJ. 1989. Mucoid phenotype of *Klebsiella pneumoniae* is a plasmid-encoded virulence factor. Infect Immun 57:546–552. doi:10.1128/iai.57.2.546-552.1989. 2643575 PMC313131

[8] Nassif X, Sansonetti PJ. 1986. Correlation of the virulence of *Klebsiella pneumoniae* K1 and K2 with the presence of a plasmid encoding aerobactin. Infect Immun 54:603–608. doi:10.1128/iai.54.3.603-608.1986. 2946641 PMC260211

[9] Mike LA, Stark AJ, Forsyth VS, Vornhagen J, Smith SN, Bachman MA, Mobley HLT. 2021. A systematic analysis of hypermucoviscosity and capsule reveals distinct and overlapping genes that impact Klebsiella pneumoniae fitness. PLoS Pathog 17:e1009376. doi:10.1371/journal.ppat.1009376. 33720976 PMC7993769

[10] Walker KA, Miner TA, Palacios M, Trzilova D, Frederick DR, Broberg CA, Sepúlveda VE, Quinn JD, Miller VL. 2019. A *Klebsiella pneumoniae* regulatory mutant has reduced capsule expression but retains hypermucoviscosity. mBio 10:e00089-19. doi:10.1128/mBio.00089-19. 30914502 PMC6437046

[11] Walker KA, Treat LP, Sepúlveda VE, Miller VL. 2020. The small protein RmpD drives hypermucoviscosity in *Klebsiella pneumoniae*. mBio 11:e01750-20. doi:10.1128/mBio.01750-20. 32963003 PMC7512549

[12] Lin T-L, Lee C-Z, Hsieh P-F, Tsai S-F, Wang J-T. 2008. Characterization of integrative and conjugative element ICE*Kp1*-associated genomic heterogeneity in a *Klebsiella pneumoniae* strain isolated from a primary liver abscess. J Bacteriol 190:515–526. doi:10.1128/JB.01219-07. 17981959 PMC2223707

[13] Wu K-M, Li L-H, Yan J-J, Tsao N, Liao T-L, Tsai H-C, Fung C-P, Chen H-J, Liu Y-M, Wang J-T, Fang C-T, Chang S-C, Shu H-Y, Liu T-T, Chen Y-T, Shiau Y-R, Lauderdale T-L, Su I-J, Kirby R, Tsai S-F. 2009. Genome sequencing and comparative analysis of *Klebsiella pneumoniae* NTUH-K2044, a strain causing liver abscess and meningitis. J Bacteriol 191:4492–4501. doi:10.1128/JB.00315-09. 19447910 PMC2704730

[14] Chen Y-T, Chang H-Y, Lai Y-C, Pan C-C, Tsai S-F, Peng H-L. 2004. Sequencing and analysis of the large virulence plasmid pLVPK of Klebsiella pneumoniae CG43. Gene 337:189–198. doi:10.1016/j.gene.2004.05.008. 15276215

[15] Yang X, Sun Q, Li J, Jiang Y, Li Y, Lin J, Chen K, Chan EW-C, Zhang R, Chen S. 2022. Molecular epidemiology of carbapenem-resistant hypervirulent Klebsiella pneumoniae in China. Emerg Microbes Infect 11:841–849. doi:10.1080/22221751.2022.2049458. 35236251 PMC8942559

[16] Hu D, Li Y, Ren P, Tian D, Chen W, Fu P, Wang W, Li X, Jiang X. 2021. Molecular epidemiology of hypervirulent carbapenemase-producing *Klebsiella pneumoniae*. Front Cell Infect Microbiol 11:661218. doi:10.3389/fcimb.2021.661218. 33898334 PMC8058458

[17] Yang X, Wai-Chi Chan E, Zhang R, Chen S. 2019. A conjugative plasmid that augments virulence in Klebsiella pneumoniae. Nat Microbiol 4:2039–2043. doi:10.1038/s41564-019-0566-7. 31570866

[18] Gu D, Dong N, Zheng Z, Lin D, Huang M, Wang L, Chan EW-C, Shu L, Yu J, Zhang R, Chen S. 2018. A fatal outbreak of ST11 carbapenem-resistant hypervirulent Klebsiella pneumoniae in a Chinese hospital: a molecular epidemiological study. Lancet Infect Dis 18:37–46. doi:10.1016/S1473-3099(17)30489-9. 28864030

[19] Choi EH, Zhang F, Lu Y-J, Malley R. 2016. Capsular polysaccharide (CPS) release by serotype 3 pneumococcal strains reduces the protective effect of anti-type 3 CPS antibodies. Clin Vaccine Immunol 23:162–167. doi:10.1128/CVI.00591-15. 26677201 PMC4744920

[20] Yang X, Xie M, Xu Q, Ye L, Yang C, Dong N, Chan EW-C, Zhang R, Chen S. 2022. Transmission of pLVPK-like virulence plasmid in Klebsiella pneumoniae mediated by an Incl1 conjugative helper plasmid. iScience 25:104428. doi:10.1016/j.isci.2022.104428. 35663037 PMC9160755

[21] Yang X, Dong N, Liu X, Yang C, Ye L, Chan EW-C, Zhang R, Chen S. 2021. Co-conjugation of virulence plasmid and KPC plasmid in a clinical Klebsiella pneumoniae strain. Front Microbiol 12:739461. doi:10.3389/fmicb.2021.739461. 34819921 PMC8606748

[22] Domenico P, Schwartz S, Cunha BA. 1989. Reduction of capsular polysaccharide production in *Klebsiella pneumoniae* by sodium salicylate. Infect Immun 57:3778–3782. doi:10.1128/iai.57.12.3778-3782.1989. 2680983 PMC259904

[23] Xie M, Chen K, Ye L, Yang X, Xu Q, Yang C, Dong N, Chan EW-C, Sun Q, Shu L, Gu D, Lin X, Zhang R, Chen S. 2020. Conjugation of virulence plasmid in clinical Klebsiella pneumoniae strains through formation of a fusion plasmid. Adv Biosyst 4:1900239. doi:10.1002/adbi.201900239. 32293159

