# Supplementary material for: Functional Characterization of Plasmid-Borne rmpADC Homologues in Klebsiella pneumoniae
Source: Microbiol Spectr. 2023 Apr 24;11(3):e03081-22. doi: 10.1128/spectrum.03081-22 (PMC10269444; doi:10.1128/spectrum.03081-22)

Table S1. Primers used in this work.

Primers	Sequences (5’-3’)
rmpA-BamHI_F	CG*GGATCC*AACCAACGACTTTCAAGAGAAATGA
rmpA-HindIII_R	CCC*AAGCTT*CTTCACATCCCCTCCCCTTTT
rmpD-BamHI_F	CG*GGATCC*GGTTGATGAAAGATGGCTCATG
rmpD-HindIII_R	CCC*AAGCTT*GGCATGAGTTATATATCGCGC
rmpC-BamHI_F	CG*GGATCC*CATCTCCAGCAAATGAGAAC
rmpC-HindIII_R	CCC*AAGCTT*GCTATACCATCCTTAATGAGCC
rmpA2-BamHI_F	CG*GGATCC*ACTACCATGCAAACACAAACACAA
rmpA2-HindIII_R	CCC*AAGCTT*ATAGTTCACCTCCTCCTCCCTT
rmpD2-BamHI_F	CG*GGATCC*CTGATGAAAAATGGTGCACATC
rmpD2-HindIII_R	CCC*AAGCTT*GAAGTAAGTGTTTTCGGTAACG

Figure S1. Mucoviscosity assay of PM8PC and the derives carrying *rmpC/D*-pSC101.

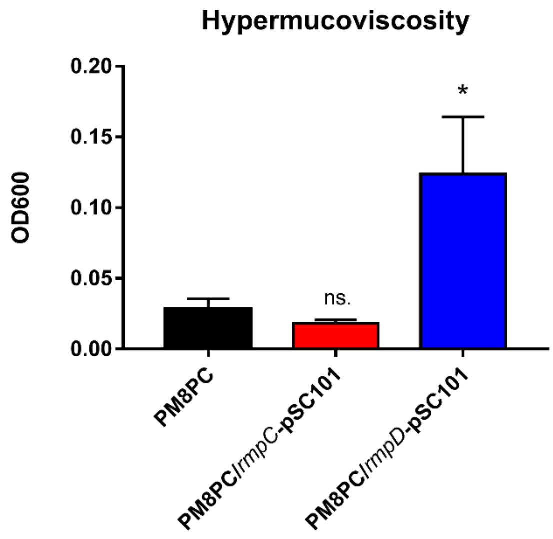

Supplement: Supplemental file 1 — Table S1 and Fig. S1. Download spectrum.03081-22-s0001.pdf, PDF file, 0.1 MB [file spectrum.03081-22-s0001.pdf]
